# Psychological trauma occurring during adolescence is associated with an increased risk of greater waist circumference in Early Psychosis patients treated with psychotropic medication

**DOI:** 10.1371/journal.pone.0242569

**Published:** 2020-12-03

**Authors:** Luis Alameda, Axel Levier, Mehdi Gholam-Rezaee, Philippe Golay, Frederik Vandenberghe, Aurélie Delacretaz, Philipp Baumann, Anaïs Glatard, Céline Dubath, Andres Herane-Vives, Victoria Rodriguez, Alessandra Solida, Kim Q. Do, Chin B. Eap, Philippe Conus

**Affiliations:** 1 Department of Psychosis Studies, Institute of Psychiatry, Psychology & Neuroscience, King’s College London, London, United Kingdom; 2 Service of General Psychiatry, Treatment and Early Intervention in Psychosis, Program [TIPP-Lausanne], Lausanne University Hospital [CHUV], Lausanne, Switzerland; 3 Department of Psychiatry, Hospital Universitario Virgen del Rocío, Universidad de Sevilla, Sevilla, Spain; 4 Instituto de Investigación Sanitaria de Sevilla, IBiS, Sevilla, Spain; 5 Unit of Pharmacogenetics and Clinical Psychopharmacology, Centre for Psychiatric Neuroscience, Department of Psychiatry, Hospital of Cery, Lausanne University Hospital, Prilly, Switzerland; 6 Department of Psychiatry, Center for Psychiatric Epidemiology and Psychopathology, Lausanne University Hospital [CHUV], Lausanne, Switzerland; 7 Community Psychiatry Service, Department of Psychiatry, Consultations de Chauderon, Lausanne University Hospital and University of Lausanne, Lausanne, Switzerland; 8 Institute of Psychology, faculty of Social and Politic al Science, University of Lausanne, Lausanne, Switzerland; 9 Unit for Research in Schizophrenia, Center for Psychiatric Neuroscience, Department of Psychiatry, Lausanne University Hospital [CHUV], Lausanne, Switzerland; 10 Department of Psychological Medicine, Affective Disorders Research Group, Centre for Affective Disorders, Institute of Psychiatry, Psychology & Neuroscience, King’s College London, London, United Kingdom; 11 Departamento de Clínicas, Facultad de Medicina, Universidad Católica del Norte, Coquimbo, Chile; 12 Affective Disorders Research Group, Centre for Affective Disorders, Department of Psychological Medicine, Institute of Psychiatry, Psychology and Neuroscience King’s College London, London, United Kingdom; 13 Department of Pharmaceutical Sciences, School of Pharmacy, University of Geneva, Geneva, Switzerland; National Autonomous University of Mexico, MEXICO

## Abstract

**Background:**

It has been suggested that exposure to Childhood Trauma [CT] may play a role in the risk of obesity in Early Psychosis [EP] patients; however, whether this is independently of age at exposure to CT and the medication profile has yet to be investigated.

**Methods:**

113 EP-patients aged 18–35 were recruited from the Treatment and Early Intervention in Psychosis Program [TIPP-Lausanne]. Body Mass Index [BMI], Weight Gain [WG] and Waist Circumference [WC] were measured prospectively at baseline and after 1, 2, 3, 6 and 12 months of weight gain inducing psychotropic treatment. Patients were classified as Early-Trauma and Late-Trauma if the exposure had occurred before age 12 or between ages 12 and 16 respectively. Generalized Linear Mixed-Models were adjusted for age, sex, socioeconomic status, baseline BMI, medication and for diagnosis of depression.

**Results:**

Late-Trauma patients, when compared to Non-Trauma patients showed greater WCs during the follow-up [p = 0.013]. No differences were found in any of the other follow-up measures.

**Conclusions:**

Exposition to CT during adolescence in EP-patients treated with psychotropic medication is associated with greater WC during the early phase of the disease. Further investigation exploring mechanisms underlying the interactions between peripubertal stress, corticoids responsiveness and a subsequent increase of abdominal adiposity is warranted.

## Introduction

Patients suffering from psychosis have higher mortality rates [1] and about 18 years shorter life-expectancy when compared to the general population [2]. Risk factors such as obesity, alongside other physical health problems [smoking tobacco, hypertension, type 2 diabetes mellitus, dyslipidemia and cardiovascular disease] play an important role in the excess of mortality in patients suffering from psychosis and other severe mental illness [3–5]. Because psychotropic medications can induce strong weight gain [WG] and other metabolic disorders [6], adapted interventions targeting the well-known iatrogenic consequences of medication and life style have been proposed in the last 15 years, with positive results [7]. However, psychotropic medication is not the only weight gain predictor and some metabolic disturbances are already present in drug naïve patients in the early phase of the psychotic disorder [Early psychosis: EP] [8–10]. Identification of other risk factors could lead to a better understanding of the biological mechanisms underlying obesity in psychotic patients and allow the implementation of new preventive interventions.

Childhood trauma [CT] is a risk factor for psychosis [11], and a meta-analysis of 41 epidemiological studies in the general population revealed that exposure to trauma was associated with an increased risk of developing obesity [odds ratio: 1.36; 95% confidence interval 1.26–1,47] [12]. One study showed that traumatized EP patients had higher BMI when compared to controls and to non-traumatized EP patients [13]. Another study showed a positive association between a number of different forms of abuse and BMI in EP patients [14] and suggests a possible interplay between increased inflammation [CRP], BMI and CT. Furthermore, both inflammation [15, 16] and CT [17] have been associated with higher levels of depressive symptoms in EP patients, and depressive patients [especially those with atypical features] are at higher risk of WG independently of medication [18]. For this reason, it is thus important to take into account depression as a potential confounder when considering the link between trauma and obesity related outcomes. Moreover, different developmental periods are important for processes involved in weight gain such as energy intake, storage and expenditure [12]. Thus, if trauma exposure influences the risk of obesity, taking into account the age at which the patient is exposed may lead to a better understanding of which developmental trajectories are operating on the link between trauma and obesity related outcomes. Considering that the studies that examined the association between CT and obesity related measures in patients with psychosis were cross sectional, a study addressing this question of the weight gain with a prospective setting is needed. This may allow the investigation into whether metabolic disturbances are already present at the beginning of the psychotropic treatment, or if they develop at a later stage.

Given the above, the present study aimed to examine in a sample of EP patient, the differential impact of trauma occurring during childhood or adolescence on the risk of obesity related outcomes during the first year of psychotropic treatment. We took into account a number of potential covariates and confounders such as a diagnosis of depression, age, sex, socio-economic status [SES], baseline BMI and medication, before and during the follow up.

## Methods

### Procedure and subjects

TIPP [Treatment and Early Intervention in Psychosis Program], a specialized early psychosis program, was launched in 2004 at the Department of Psychiatry, Lausanne University Hospital Switzerland [19]. Entry criteria to the program are: [I] aged between 18 and 35; [II] residing in the catchment area [Lausanne and surroundings; population about 300 000]; [III] meeting threshold criteria for psychosis, as defined by the “Psychosis threshold” subscale of the Comprehensive Assessment of At Risk Mental States [CAARMS [20]] Scale [19]. In the TIPP program, each patient is followed up by a psychiatrist and a Case manager. The program offers an integrated biopsychosocial treatment based on psychotherapy, psychoeducation, family support, cognitive assessment and remediation [when needed], social support, assistance in finding employment, psychological interventions for cannabis use, and pharmacological treatment. A specially designed questionnaire [21] is completed for all patients enrolled in the programme by case managers who have up to 100 contacts with patients during the 3 years of treatment. It allows assessment of demographic characteristics, past medical history, exposure to life events as well as symptoms and functioning. It is completed on the basis of information gathered with patients and their family over the first few weeks of treatment and can be updated during follow-up if new information emerges. Follow-up assessments exploring various aspects of treatment and co-morbidities as well as evolution of psychopathology and functional level are conducted by a research psychologist and by case managers after 2, 6, 12, 18, 24, 30 and 36 months in treatment. In particular, substance abuse diagnosis was assessed at each time-point and rated on the basis of DSM-IV criteria [22].

Patients starting a pharmacological treatment that is known to have a potential risk to induce metabolic disturbances [i.e. all antipsychotics, some antidepressants and mood stabilizers] are prospectively followed up for metabolic parameters evolution [see Biological and physical measures section below] [21, 23]. Since 2007, a study has been ongoing in the Department of Psychiatry of the Lausanne University Hospital using the routinely collected data to better characterize and understand metabolic adverse effect onset [PsyMetab-PsyClin].

Capacity to consent was determined by the case manager involved in the clinical care of the patient. This ensures that each patient has capacity when they are asked to give consent to participate in the study. If the patient is temporarily unable to give an informed consent, research questions are not presented, until his capacity to consent is recovered. In case patients are evaluated as having a permanent lack of capacity for understanding the research questions, the consent of a legal representative is asked. The Ethics Committee of the Canton of Vaud granted access to TIPP clinical data [demographic, assessment of trauma history and levels of symptoms and other clinical variables] an also granted access to physical data [obesity-related outcomes] as part of PsyMetab cohort.

Patients included in the PsyMetab cohort were requested to give their written informed consent to participate in the study and to have their data to be used for research studies. In addition, up to end of 2015,] clinical data from patients from PsyClin could be used for the current study as part of non-interventional post hoc analysis. Both PsyMetab-PsyClin protocols, as well as this particular study, were approved by the Ethics Committee of the Canton of Vaud [CER-VD].

The present analysis involves participants included in both the TIPP program and who were also part of the PsyMetab-PsyClin study between 2007 and end of 2016.

### Assessment of history of past trauma

Clinicians at TIPP are trained to conduct an extensive assessment of patients, including evaluation of exposure to traumatic life events. Case managers meet patients frequently over the treatment period, which provides the framework to establish a trusting relationship, where extensive knowledge of patients’ history can be gathered. If patients agree, information can also be completed with family. In the case of inconsistency between the patient’s and the family’s report or in case of doubt regarding the exposure to trauma or the age at the time of exposure, patients were not included in the study [24]. Case managers complete a table during the patients’ 3 years of treatment, where exposure to traumatic life events can be recorded as follows: [1] Type of traumatic life event, rated as present or absent [sexual abuse, physical abuse, emotional and physical neglect, emotional abuse, among others…]; [2] time of occurrence in relation to psychosis stage [during the premorbid phase, during the prodrome or after onset of psychosis]; [3] age at the time of first exposure to each one of the traumas that occurred; and [4] single or repeated exposure to each one of the traumas that occurred. Considering that the clinicians who assessed exposure to life events did not rate the subjective perception of severity of the different forms of stressful events, patients were considered traumatized if they had been exposed to at least one experiences of abuse [physical, sexual, or emotional] or neglect [physical or emotional]. The consideration was that such events would undoubtedly be considered as highly traumatizing by anyone, and have been shown to be associated with risk for psychosis and functional deficits in psychotic samples [25]. Sexual abuse refers to sexual molestation and/or rape. Physical abuse refers to physical attack or assault, or being repetitively beaten by parents, relatives, or caregivers. Emotional abuse was defined as verbal assaults on a child’s sense of worth or well-being or any humiliating or demeaning behaviour directed toward a child by an adult or older person. Physical neglect was defined as the failure of caretakers to provide for a child’s basic physical needs, including food, shelter, clothing, safety, and health care. Emotional neglect was defined as the failure of caretakers to meet children’s basic emotional and psychological needs, including love, belonging, nurturance, and support. Age at the time of first exposure was categorized as follows [1] early trauma refers to exposure between birth and age 12, according to conventions applied elsewhere [26–28] late trauma refers to exposure between ages 12 and 16. Patients who were exposed to trauma after age 16 were excluded from this study, according to other studies [17, 24] suggesting that they may already have been in the prodromal phase of their first psychotic episode.

### Biological and physical measures

Data of patients in the PsyMetab-PsyClin studies were collected during hospitalization and/or in outpatient centers. Following the introduction of psychotropic treatments that have been linked to weight gain, [weight gain inducing psychotropic medications], a prospective monitoring [at times 0, 1, 2, 3, 6, 9 and 12 months] of body mass index [BMI], waist circumference [WC] and weight gain [WG] was performed. In the case of an interruption of the above-mentioned weight gain inducing psychotropic medication for more than two weeks or in the case of replacement by another drug, the follow-up is restarted from baseline. In the case of an introduction of a second drug from the list [see list below], the follow-up is restarted. In this study, we considered only the earliest follow-up which has less missing weight information.

### Other clinical variables

Main diagnosis and comorbid diagnosis of depression were based on DSM-IV [22] criteria according to others [29]. Medications were categorized into low, medium and high-risk of weight gain as follows [30] [High: Valproate, Olanzapine and Clozapine; Moderate: Quetiapine, Risperidone, Paliperidone, Lithium, Mirtazapine, Zuclopenthixol, Levomepromazine; Low: Amisulpride, Aripiprazole, Haloperidol, Lurasidone, Flupentixol]. In order to adjust by medication intake, we created two variables: [i] Medication during follow-up, which refers to the medication taken during the 1 year of follow-up, and which was categorized into High, Moderate and Low risk; [ii] Medication before the first assessment, which refers to the medication that patients had taken before being included in this study. This variable was categorized as 0 [no psychotropic treatment before the first assessment] and 1 [prescription of at least one of the medications from the list [see above] before the first assessment, with partial or total adherence]. Estimation of the adherence was based on the Treatment Adherence Scale [TAS] [31], which is assessed by the case manager based on results from the plasmatic concentration measured routinely, interviews with patients and an extensive knowledge of the patient treatment and adherence with medication and service. This is evaluated at every TIPP follow-up time points and which is scored on the basis of a 3 point scale [0 = non adherence; 1 = partial adherence, from 25% to 75% of the time during the evaluation period; and 2 = total adherence, from 75% to 100% of the time during the evaluation period]. Patients were considered as adherent to treatment if they scored 2 on the TAS. Socio-economic status [SES] were subdivided into high, intermediate and low based on the socio-economic status of the parents as previously described [32].

### Statistical analysis

In order to test the distribution of continuous variables between groups we used the Kruskal Wallis test which is a non-parametric and robust test. This test uses the observed ranks of observations instead of their observed value and is unaffected by outliers if there is any. Chi-squares test of independence was employed to detect associations between categorical variables and the trauma group [trauma], considering a level below alpha = 0.05 as enough evidence to reject the independence between categorical variables and trauma group. [If the test is rejected in the level of alpha = 0.05 then we have enough evidence to reject the independence between the categorical variable and the group.] This test is valid unless the expected frequency is smaller than 5 in at least 20% of cells, when the Fisher Exact Test should be used instead. Generalized Linear Mixed Models [GLMMs] were used to measure the effect of trauma on the response variables, adjusting the models for potential covariates and confounders [diagnosis of depression, age, sex, socio-economic status [SES], baseline BMI and medication before and during the follow up [as detailed in previous section]]. The main advantage of GLMMs is the presence of random effects that allows adjustment of the model for interdependence among observations. For all models, we have introduced a common random intercept for observations corresponding to each individual. A Bayesian estimation approach with flat priors was used to adjust these models, due to its convenience in dealing with models of this complexity. To do so, we used a large number of Markov Chain Monte-Carlo iterations [10 million], observing satisfactory convergence criteria for all models. These models were adjusted using the MCMCglmm package [33] developed for R [34] environment for statistical computing. Statistical significance was determined by a P value <0.05.

## Results

### Patient sample

Among the 143 EP patients who accepted to participate in the study, 30 were excluded for the following reasons: [I] age at exposure to trauma was not available or there was a doubt about the date at the time of exposure [n = 4]; [II] first exposure to trauma occurred during the prodromal phase or after psychosis onset [n = 3]; [III] the physical measures were done more than 5 years after the entry into TIPP program [n = 23]. Therefore, analysis was carried out with the data of the remaining 113 patients.

### Rates of trauma, demographics and baseline biological and physical measurements

The diagnostic breakdown, baseline demographic and clinical information including diagnosis alongside with biological measures of the sample are described in Table 1.

**Table 1:** Rates of trauma, demographics and baseline physical measurements.

	Total N = 113	Non Trauma N = 74	Early Trauma N = 28	Late Trauma N = 11	P-value
Age, median [range], years	25 [18–39]	25 [18–37]	27.5 [19–37]	24 [19–39]	0.558
Female, N [%]	36 [31.9]	21 [28.4]	13 [46.4]	2 [18.8]	0.12
Smoking, N [%]	46 [40.7]	31 [41.9]	11 [39.3]	4 [36.4]	0,955
SES, N [%]					
Low	23 [20.4]	18 [24.3]	4 [14.3]	1 [9.1]	0.092
Medium	47 [41.6]	33 [44.6]	12 [42.9]	2 [18.2]	
High	43 [38.0]	23 [31.1]	12 [42.9]	8 [72.7]	
Diagnostic, No [%]					
Schizophrenia	70 [61.9]	45 [60.8]	19 [67.9]	6 [54.5]	0.462
Schizophreniform/BPE	10 [8.9]	6 [8.1]	4 [14.3]	0 [0.0]	
Schizoaffective disorder	12 [10.6]	9 [12.2]	0 [0.0]	3 [27.3]	
Major depression with PF	4 [3.5]	3 [4.0]	1 [3.6]	0 [0.0]	
Bipolar Disorder	8 [7.1]	6 [8.1]	1 [3.6]	1 [9.1]	
Others	9 [8.0]	5 [6.8]	3 [10.7]	1 [9.1]	
Trauma subtypes					
Sexual abuse	13 [11.5]	-	9 [32.1]	4 [36.4]	0.801
Physical abuse	18 [16]	-	13 [46.4]	5 [45.5]	0.956
Emotional abuse	5 [4.4]	-	3 [10.7]	2 [18.2]	0.530
Physical neglect	5 [4.4]	-	4 [14.3]	1 [9.1]	0.662
Emotional neglect	10 [8.8]	-	8 [28.6]	2 [18.2]	0.503
Treatment prior first assessment N [%]	54 [47.8]	33 [44.6]	13 [46.4]	8 [72.7]	0.216
Treatment during follow up* N [%]					
Low	28 [24.8]	17 [23.0]	7 [25.0]	4 [36.4]	0.236
Moderate	56 [49.6]	40 [54.0]	10 [35.7]	6 [54.5]	
High	29 [25.7]	17 [23.0]	11 [39.3]	1 [9.1]	
Baseline physical measures #					
BMI, median [range] kg/ m^2^	21.9 [15.6–35.4]	22.5 [15.6–33.6]	20.9 [16.8–35.4]	21.4 [17.5–30.1]	0.453
Overweight, N [%]	21 [19.6]	15 [21.7]	3 [11.1]	3 [27.3]	0.723
WC, median [range], cm	84 [45–114]	83 [58–114]	79 [45–110]	93 [82–110]	0,051
High WC, N [%] £	21 [24.1]	13 [22.4]	4 [19.1]	4 [50.0]	0.191

SES: Socio-economic-status; N = number of patients; PF: psychotic features; BPE: brief psychotic episode; BMI: Body Mass Index; WC: wait circumference

* treatment was categorized as function of risk of gaining weight into High: valproate, olanzapine and clozapine; Moderate: Quetiapine, Risperidone, Paliperidone, Lithium, Mirtazapine, Zuclopenthixol, Levomepromazine; Low: Amisulpride, Aripiprazole, Haloperidol, Lurasidone, Flupentixol;

^#^25 kg/m2 ≥ Initial BMI<30 kg/m2, %;

^£^:High WC ≥ 94 cm [male], ≥ 88 cm [female] Early Trauma: refers to exposure from birth through 11 years of age; Late Trauma: refers to exposure between from age 12 years through 16 years.

Among 113 patients, 39 [34.5% of the total] had a history of at least one experience of trauma. Within the group with trauma, 28 [71.7% of exposed patients] had been exposed before age 12 [Early-trauma] and 11 [28.3% of exposed patients] between 12 and 16 [Late-trauma]. There were no differences in the rates of exposure to each individual traumatic experience [sexual, physical and emotional abuse; physical and emotional neglect] between patients exposed in childhood or in adolescence.

At baseline, no differences between Early-trauma, Late-trauma and Non-Trauma patients in terms of diagnostic distribution, levels of overweight, BMI, age, sex, SES, smoking status and the presence of treatment prior first assessment and during follow-up were observed.

### Association of Early- and Late-Trauma with the BMI, weight gain and waist circumference during follow-up

**Fig 1:**
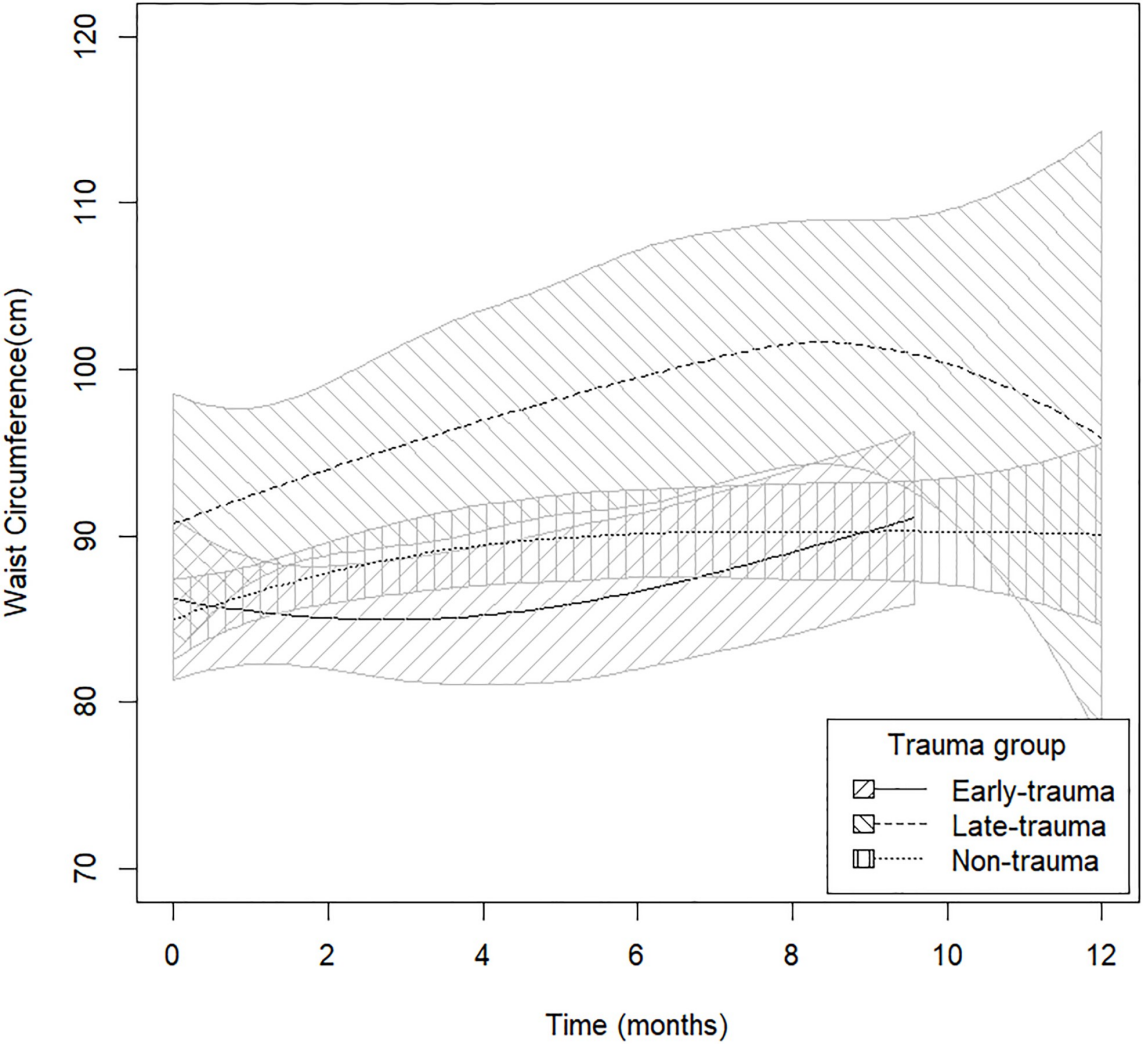
Graphical illustration of the differences in the waist circumference during follow-up [0, 2, 4, 6, 8, 10 and 12 months] between non trauma, Early Trauma and Late Trauma patients. The plot intends to capture a smooth trend in waist circumference for each subgroup of individuals using a spline fitted separately to each group. For each plot 95% Confidence bounds are also added to the plot. The plot is not adjusted for potential covariates but is rather a graphical mean to explore the variation of waist-circumference in function of time.

Detailed results of GLMM models comparing the BMI, WC and WG at follow-up between Early, Late-Trauma and Non-Trauma are given in Table 2 and represented in Fig 1.

**Table 2:** BMI, weight gain and waist circumference levels of patients exposed to Early and Late Trauma compared with nonexposed patients across 1 year follow-up.

	Early Trauma			Late Trauma		
	Estimate	CI [95%]	P value	Estimate	CI [95%]	P value
BMI, Kg/ m^2^	0.139	[-0.62–0.89]	0.718	0.178	[-0.90–1.27]	0.742
Weight Gain in Kg	0.193	[-3.04–3.38]	0.906	0.562	[-3.99–5.17]	0.806
Waist Circumference, in cm	-1.626	[-4.67–1.41]	0.295	5.545	[1.14–9.85]	0.013*

BMI: Body Mass Index; CI: confidence interval. Early Trauma: refers to exposure from birth through 11 years of age; Late Trauma: refers to exposure between from age 12 years through 16 years.

* Significant at the 0.05 level.

Compared to Non-Trauma patients, Early-Trauma patients did not differ in terms of BMI, WG and WC during follow-up. Compared to Non-Trauma patients, Late-Trauma patients did not differ in terms of BMI and WG during follow-up. However, they showed higher levels of WC [*β* = 5.545; p = 0.013] during follow-up. Results related to potential confounders and covariates are shown in S1 Table, being time in treatment and baseline BMI the only variables significantly associated with BMI, WG and WC.

## Discussion

We found that EP patients with trauma occurring between 12 and 16 years of age display higher WC during the first year of psychotropic medication compared to patients without trauma. In contrast, patients exposed to trauma before age 12 displayed similar levels of obesity related outcomes than non-traumatized patients. Our results support evidence suggesting that severe stress during adolescence could contribute to increased waist circumference in patients with psychosis [13, 14]. This suggests that adolescence is a critical period at which stress can increase the vulnerability, in combination with other factors, to a later increase in WC, and subsequent obesity, which opens new venues for potential mechanisms explaining this link.

These results accounted for age, sex, diagnosis of depression, presence of psychotropic prescription before entry into the program and class of risk of medication intake during follow up. We did not find any association between exposure to trauma, neither during childhood nor adolescence, and increased BMI or weight change during follow-up, which suggests a stronger association of peripubertal psychological stress with abdominal adiposity.

The positive association between trauma and risk of obesity related outcomes has previously been reported in non-clinical samples [12]. Two studies have already shown an association between CT and greater BMI in patients with EP [13, 14]. Our study does not replicate the results regarding BMI, which could be due to a question of sample size. It may be possible that patients develop firstly a greater WC, and this contributes to later obesity and other cardiovascular risk factors. However, our stratification of traumatized patients according to the age at first exposure highlights a new finding: only patients exposed to trauma during adolescence display higher WC during the follow-up when compared to non-traumatized patients, being this association independent of a broad range of potential confounders. These results not only highlight the importance of stratifying patients according to the age of exposure but also open new venues for potential different neuro-developmental stages operating on the link between trauma and obesity related outcomes.

The Hypothalamus Pituitary Adrenal [HPA] axis is the most important regulator of the stress response [35] and stands as one of the potential mediating pathways linking trauma to psychopathology [36], including psychosis [11]. Interestingly, evidence from clinical studies in adolescent subjects to cellular and molecular studies show that elevated cortisol, particularly when combined with inhibition of sex steroids and growth hormone secretions, is causing accumulation of fat in visceral adipose tissues as well as metabolic abnormalities [37–39].

Moreover, teenagers, as a result of the pubertal surge in sex steroids, have shown increased responsiveness to stress hormones, leading to greater stress induced HPA axis responses as compared with adults [40, 41]. Taken altogether, we could speculate that trauma occurring during adolescence may induce elevated levels of glucocorticoids which, combined with inhibition of sex steroids, would lead to increased fat accumulation in visceral adipose tissue, contributing to a greater WC.

Contrary to what has been found by others [42, 43], we did not find that a diagnosis of depression accounted for changes in any of the obesity related outcomes. However, whether the depression [or sub-diagnostic depressive symptoms and different depression subtypes [44]] mediates the link between trauma and obesity related outcomes in psychosis needs to be explored in larger prospective samples. We did not find either an association between medication before the first assessment or during follow-up with our outcomes. This could be explained in part by a beneficial impact of the preventive strategies proposed by clinicians in charge of patients receiving psychotropic drugs at risk of weight gain that are part of the prospective monitoring implemented in the Lausanne University hospital [45]. Indeed, this follow up aims to reduce the negative impact of medication on weight and on metabolic parameters. These preventive measures include: [i] linking with general practitioner in the follow-up in order to activate a rapid response in case of weight gain or when an alteration in any of the metabolic disturbances appear; [ii] a continuous risk-benefit balance evaluation by the clinician in charge, considering switching to another medication appropriate to the clinical context every time a patient gains weight rapidly after initiation of a new psychotropic medication [iii] encouraging of patients to undertake healthy lifestyle habits, such as doing sport, yoga or being monitored by a nutritionist in case of rapid weight gain, especially during the first weeks of treatment [46].

Our study has various limitations. Firstly, although the obesity related outcomes were collected prospectively, exposure to trauma was assessed retrospectively, which may be particularly problematic for patients suffering from psychosis due to recall bias [47]. Moreover, although we looked at the time of exposure, we did not take into account other possible aspects such as the duration of the exposure, which may have an important modulating effect on out measures outcomes. Despite these limitations, exposure to trauma was assessed by the case manager who followed each patient on the basis of information obtained from patients [48] and their families in the context of a 3-year therapeutic relationship. This allowed the clinician to choose the most appropriate moment when the patient did not present a mental state that could affect the recall of those experiences and may help some patients to acknowledge some experiences such as sexual abuses. Second, As we only followed patients after their first episode of psychosis and the lower age to be included in TIPP is 18, we cannot exclude the possibility that actually the increased peri abdominal adiposity in people exposed to trauma in adolescence was present prior the exposure to trauma, thus our study design does not infer causality and prospective studies following individuals from childhood to adulthood can provide a confirmation on our results. Secondly, many patients of the TIPP cohort refused to take part in the prospective metabolic follow-up, and we cannot exclude a selection bias in our sample [with patients not included in this study displaying different obesity related outcomes according to trauma exposure]. Thirdly, the sample size was relatively small and thus we cannot exclude that the lack of association between CT and BMI was due to a lack of statistical power. Furthermore, other potential confounders may have played a role in our results such as physical exercise or other aspects of psychopathology, such as anxiety. Other larger studies replicating our findings, including patients without psychotropic medication and a healthy control group exposed to CT are needed.

In conclusion, this is the first study showing that patients exposed to trauma during adolescence and being treated with AP medication have an increased WC during the early phase of the disease, independently of factors such as the previous medication or depression. These results open new venues toward possible mechanisms underlying the link between peripubertal stress and peri-abdominal adiposity, such as the possible impact of glucocorticoids related to a dysregulation of the HPA axis during the adolescence after exposure to CT. Given the high rates of cardiovascular-related mortality of patients with psychosis, there is an urgent need to further understand the association between trauma and obesity in this vulnerable population. Special attention should be paid to EPP who have been traumatized during adolescence when initiating a treatment to try to prevent an increase of WC, which is a major risk factor for metabolic syndrome [49].

## Supporting information

S1 Table Association between cofactors and confounders with BMI, weight gain and waist circumference across 1 year follow-up. (DOCX)
