# Supplementary material for: Psychological trauma occurring during adolescence is associated with an increased risk of greater waist circumference in Early Psychosis patients treated with psychotropic medication
Source: PLoS One. 2020 Dec 3;15(12):e0242569. doi: 10.1371/journal.pone.0242569 (PMC7714104; doi:10.1371/journal.pone.0242569)
Supplement: S1 Table — (DOCX) [file pone.0242569.s001.docx]

**S1 Table** Association between cofactors and confounders with BMI, weight gain and waist circumference across 1 year follow-up.

|  | BMI | | | WG | | | WC | | |
| --- | --- | --- | --- | --- | --- | --- | --- | --- | --- |
|  | Est | CI [95%] | P value | Est | CI [95%] | P value | Est | CI [95%] | P Value |
| Age [per 1 year] | -0.05 | [-0.12-0.01] | 0.114 | -0.22 | [-0.52-0.03] | 0.10 | 0.06 | [-0.21-0.33] | 0.63 |
| Sex, male | -0.29 | [-1.00-0.46] | 0.436 | -1.11 | [-4.13-2.05] | 0.47 | 1.65 | [-1.29-4.57] | 0.26 |
| Medication prior first assessment | 0.06 | [-0.64-0.79] | 0.859 | 0.17 | [-2.86-3.20] | 0.90 | -0.76 | [-3.69-2.24] | 0.61 |
| Depression | -1.64 | [-0.89-0.86] | 0.968 | -0.41 | [-4.12-3.28] | 0.82 | -1.64 | [-5.15-1.84] | 0.35 |
| Medication during FU Medium | 0.20 | [-0.58-0.99] | 0.61 | 0.90 | [-2.45-4.22] | 0.59 | 0.91 | [-2.35-4.16] | 0.57 |
| Medication during FU^&^ | 0.36 | [-0.60-1.34] | 0.457 | 1.75 | [-2.32-5.87] | 0.39 | 0.71 | [-3.26-4.73] | 0.72 |
| Time during FU [per 1 month] | 0.19 | [0.15-0.23] | <1e-05 | 0.87 | [0.69-1.06] | <1e-05 | 0.50 | [0.32-0.68] | <1e-05 |
| SES medium | -0.10 | [-0.94-0.75] | 0.798 | -0.33 | [-3.89-3.27] | 0.85 | 2.55 | [-0.90-6.02] | 0.14 |
| SES high | -0.05 | [-0.97-0.88] | 0.902 | -0.11 | [-4.04-3.81] | 0.95 | 1.52 | [-2.18-5.27] | 0.42 |
| Baseline BMI  [per 1 Kg/ m^2]^ | -0.43 | [-0.80-0.06] | <1e-05 | 2.01 | [0.80-0.06] | 0.02* | 2.01 | [1.67-2.37] | <1e-05 |

BMI: Body Mass Index [in Kg/ m^2]^; WC: wait circumference [in cm]; WG: weight gain [in Kg]; FU: follow-up; SES: Socio- economic status; Est: estimate. ^&^High: patients taking one of the following: valproate, olanzapine and clozapine. * Significant at the 0.05 level
